# Association between the triglyceride-glucose index and subclinical left ventricular systolic dysfunction in obese patients

**DOI:** 10.1186/s12933-024-02253-8

**Published:** 2024-05-07

**Authors:** Guang-an Li, Jun Huang, Jing Wang, Li Fan

**Affiliations:** 1 Department of Echocardiography, The Affiliated Changzhou Second People’s Hospital with Nanjing Medical University, 213003 Changzhou, China; 2 Department of Weight Loss Metabolic Surgery, The Affiliated Changzhou Second People’s Hospital with Nanjing Medical University, 213003 Changzhou, China

**Keywords:** Triglyceride-glucose index, Obese, Global longitudinal strain

## Abstract

**Background:**

The association between the triglyceride–glucose (TyG) index and subclinical left ventricular (LV) systolic dysfunction in obese patients remains unclear. This study aimed to investigate the relationship between the TyG index and LV global longitudinal strain (GLS) in obese patients.

**Methods:**

A total of 1028 obese patients from January 2019 to January 2024 were included in the present study. Clinical parameters and biochemical and echocardiographic data were obtained from the participants. LV GLS was obtained from the GE EchoPAC workstation for evaluating subclinical LV function. The TyG index was calculated as Ln (fasting TG [mg/dL] × fasting glucose [mg/dL]/2). LV GLS was compared between obese patients with a high TyG index and those with a low TyG index.

**Results:**

Obese patients with a high TyG index had greater incidences of hypertension, diabetes mellitus and hyperlipidaemia. The LV GLS was significantly lower in the high TyG index group than in the low TyG index group (*P* = 0.01). After adjusting for sex, age, body mass index, heart rate, hypertension, diabetes mellitus, dyslipidaemia, blood urea nitrogen, serum creatinine, LV mass and LV hypertrophy, the TyG index remained an independent risk indicator related to an LV GLS < 20% (OR: 1.520, 95% CI: 1.040 to 2.221; *P* = 0.031).

**Conclusions:**

We concluded that an increase in the TyG index is independently associated with subclinical LV systolic dysfunction in obese patients.

## Introduction

The worldwide prevalence of obesity has risen dramatically over the past two decades [1]. The World Health Organization (WHO) defines obesity as a pathological condition characterized by excessive fat accumulation [2]. Obesity is associated with various diseases, such as hypertension, type 2 diabetes mellitus (T2DM), metabolic syndrome, obstructive sleep apnoea syndrome, and cardiovascular diseases [3]. “Obesity cardiomyopathy”, which develops independently of hypertension, coronary heart disease and other heart diseases, has received increasing attention from researchers because it can cause haemodynamic alterations that predispose individuals to changes in cardiac morphology and ventricular function [4]. Metabolic disturbances, including insulin resistance, abnormal glucose transport, free fatty acid spillover, lipid toxicity, and amino acid derangement, are considered among the aetiologies of obesity-related cardiomyopathy [1].

Insulin resistance is a pathological-physiological state characterized by decreased insulin sensitivity in peripheral tissues [5]. The triglyceride–glucose (TyG) index, calculated as Ln (fasting TG [mg/dL] × fasting glucose [mg/dL]/2), has emerged as a new and credible indicator of insulin resistance, and some studies have indicated that the TyG index is associated with coronary artery disease [6], chronic kidney disease [7, 8], hypertension [9], T2DM [10], atrial fibrillation [11], etc.

Left ventricular (LV) global longitudinal strain (GLS) derived from two-dimensional speckle tracking echocardiography (STE) has been confirmed to accurately detect LV systolic dysfunction in various cardiovascular diseases, such as coronary artery disease [12, 13], hypertension [14], and T2DM [15]. However, the association between the TyG index and LV GLS in obese patients is still unknown.

In this study, we used STE technology to determine the associations between the TyG index and LV GLS in obese patients and between the TyG index and myocardial systolic dysfunction during the subclinical stage.

## Subjects and methods

### Ethical statement

This research was approved by the Human Research and Ethics Committee of the Affiliated Changzhou Second People’s Hospital of Nanjing Medical University. All patients completed the informed consent forms.

### Study population

**Fig. 1:**
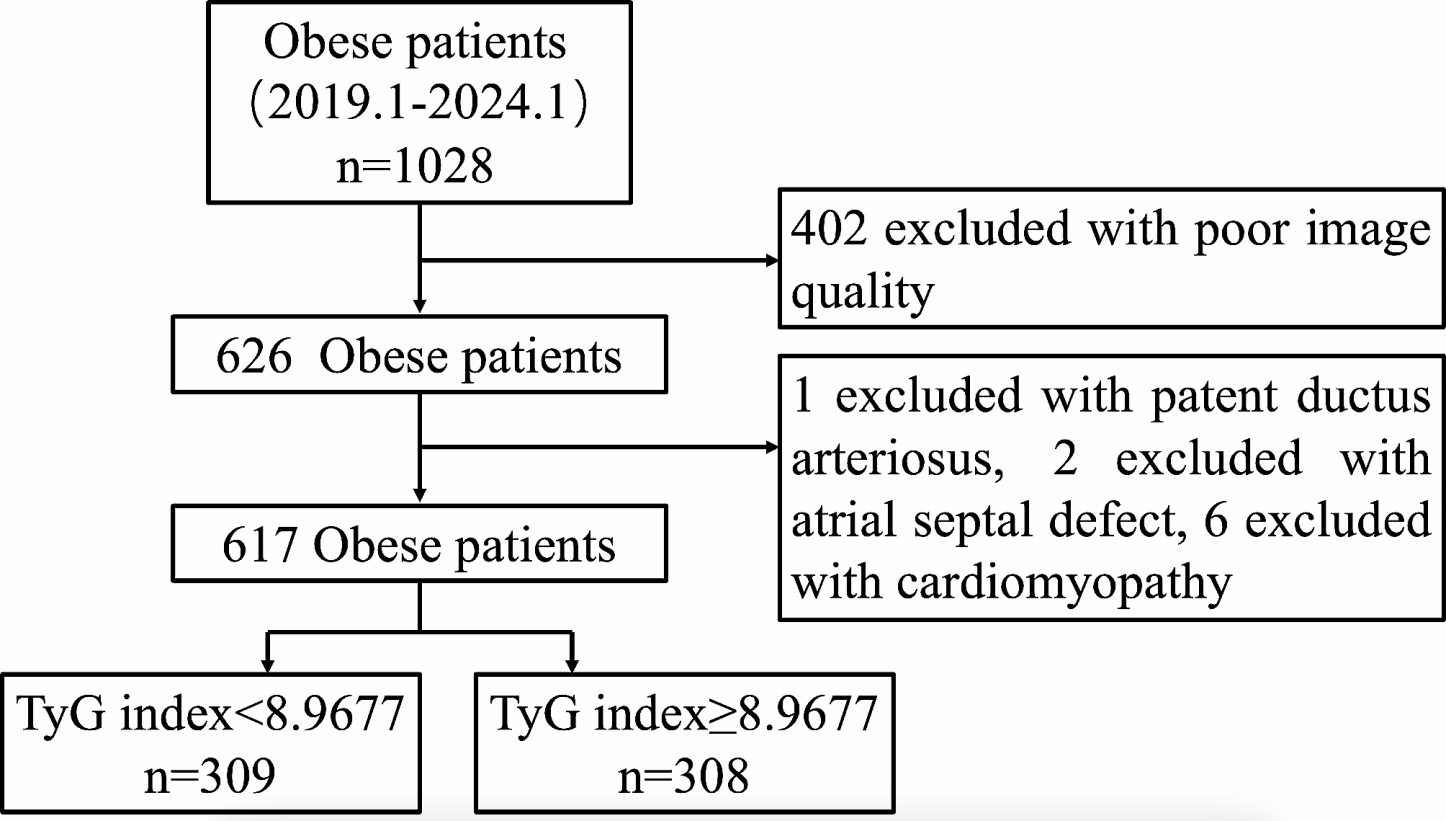
Flowchart of the selection of obese patients

This was a retrospective study. We initially included 1028 patients with obesity who underwent sleeve gastrectomy at the Department of Weight Loss Metabolic Surgery. The exclusion criteria included poor image quality, cardiomyopathy (including hypertrophic cardiomyopathy and dilated cardiomyopathy), and congenital heart disease (including atrial septal defect and patent ductus arteriosus). Patients with arrhythmia (including atrial fibrillation, premature contraction, etc.), coronary heart disease, heart failure with preserved ejection fraction (HFpEF) or valvular disease were not included in the research. All enrolled subjects had a normal ejection fraction: 52–72% for men and 54–74% for women [16] (Fig. 1).

We recorded age, sex, resting heart rate, height, weight, waist circumference, and blood pressure before echocardiography examination for all enrolled subjects. Body mass index (BMI) and body surface area (BSA) were subsequently calculated. Complications such as hypertension, diabetes mellitus and hyperlipidaemia were also recorded for each participant. The diagnoses of hypertension, diabetes mellitus and dyslipidaemia were determined according to the 2018 ESC/ESH Guidelines for the Management of Arterial Hypertension [17], the American Diabetes Association [18] and the Clinical Practice Guidelines on Hypertriglyceridaemia from the Endocrine Society [19].

Laboratory tests for fasting plasma glucose (FPG), fasting insulin, HbA1c, total cholesterol (TCH), triglyceride (TG), high-density lipoprotein (HDL-C), low-density lipoprotein (LDL-C), blood urea nitrogen (BUN) and serum creatinine (SCR) were performed when the patients were in the hospital. The TyG index was subsequently calculated as follows: Ln (fasting TG [mg/dL] × fasting glucose [mg/dL]/2). The HOMA-IR was calculated as follows: fasting insulin (µU/mL) × fasting plasma glucose (mmol/L)/22.5.

### Transthoracic echocardiography parameters

All patients underwent standard transthoracic echocardiography via a GE Vivid E9 ultrasound diagnostic system equipped with an M5s 3.5–5 MHz transducer (GE Vingmed Ultrasound, Horten, Norway) by experienced sonographers. ECG leads were connected to each patient. Two-dimensional, colour Doppler, and pulsed-wave Doppler data and standard high frame rates (> 60/s) of the apical 3-, 4- and 2-chamber views of three consecutive cycles were stored for offline analysis (EchoPAC Version: 204, GE Vingmed Ultrasound, Norway).

Septal thickness, posterior wall thickness, LV diameter (LVd), and mitral annular plane systolic excursion (MAPSE) were measured via the M-mode. Then, the relative wall thickness, LV mass, LV mass index, and LVH were calculated. The left ventricular end-diastolic volume (LVEDV), left ventricular end-systolic volume (LVESV) and left ventricular ejection fraction (LVEF) were determined via the biplane Simpson’s method. The left atrial volume (LAV) was measured by averaging the values in the apical 4- and 2-chamber views, and then the LAV index (LVMI) was calculated. The peak early and late diastolic mitral annular velocities (E and A, respectively) were measured by pulsed-wave Doppler, and the E/A ratio was then calculated. The peak early (e′) and late (a′) diastolic annular velocities were obtained by averaging the values at the septum and lateral positions using TDI, and then E/e′ was calculated.

### Two-dimensional speckle tracking echocardiography analyses

The apical 3-, 4- and 2-chamber views of three consecutive cycles were processed using the acoustic-tracking dedicated software EchoPAC (EchoPAC Version: 204, GE Vingmed Ultrasound, Norway) to estimate the LV GLS.

### Statistical analysis

According to the median value of the TyG index, patients with obesity were divided into two groups: the TyG index < 8.9677 group and the TyG index ≥ 8.9677 group. The normality of all of the values was assessed by the Shapiro‒Wilk test. Differences between two groups were compared with independent sample *t* tests for normally distributed continuous variables and are expressed as the mean ± standard deviation. The Kruskal‒Wallis rank sum test was used for nonnormally distributed continuous variables, and the results are expressed as medians (interquartile ranges). The categorical variables are presented as frequencies and percentages (%) and were compared using the chi-square test.

Correlation tests were used to assess the correlations between potential risk factors (age, sex, HR, BMI, SBP, FPG, HbA1c, TC, TG, HDL-C, LDL-C, BUN, SCR, TyG index, LV mass and LVH) and LV GLS.

Five forced-entry logistic regression models were used to determine the independent association of a GLS < 20% with the TyG index: Model 1 was the crude model, Model 2 (a multivariable model) was adjusted for age and sex, Model 3 (a multivariable model) was further adjusted for BMI and HR, Model 4 (a multivariable model) was further adjusted for hypertension, diabetes mellitus, dyslipidaemia, blood urea nitrogen, and serum creatinine, and Model 5 (a multivariable model) was further adjusted for LV mass and LVH. All of the data analyses were performed using SPSS 25.0 software (SPSS, Chicago, IL, USA). A P value < 0.05 was considered to indicate statistical significance in all tests.

### Reproducibility and repeatability

Intraobserver and interobserver variabilities in the LVGLS were determined by repeating measurements in 20 randomly selected obese patients.

## Results

We initially included 1028 patients with obesity, and 411 were excluded (402 with poor image quality, 1 with patent ductus arteriosus, 2 with atrial septal defects, and 6 with cardiomyopathy). Therefore, a total of 617 obese patients (mean age: 31.38 ± 7.48 years, male: 25.77%) were included in the present study. A total of 309 patients had a TyG index < 8.9677 (defined as the low TyG index group), and 308 had a TyG index ≥ 8.9677 (defined as the high TyG index group).

### Clinical characteristics (Table 1)

**Table 1:** Clinical parameters of obese patients between low TyG index and high TyG index

Clinical parameters	*n*	Total (*n* = 617)	*n*	Low TyG index (*n* = 309)	*n*	High TyG index (*n* = 308)	*P* value
Age, year	617	31.38 ± 7.48	309	30.07 ± 7.39	308	32.90 ± 7.60	< 0.001
Male, n (%)	617	159 (25.77)	309	54 (17.48)	308	105 (34.09)	< 0.001
Height, cm	617	166.92 ± 8.13	309	166.55 ± 7.88	308	167.70 ± 8.13	0.074
Weight, kg	617	105.05 ± 20.70	309	103.82 ± 19.53	308	106.80 ± 21.33	0.072
BMI, kg/m^2^	617	37.48 ± 5.39	309	37.24 ± 5.15	308	37.74 ± 5.52	0.248
BSA, m^2^	617	2.21 ± 0.30	309	2.19 ± 0.29	308	2.24 ± 0.31	0.060
Waist, cm	415	116.74 ± 13.67	226	116.35 ± 13.49	189	117.22 ± 13.91	0.519
SBP, mmHg	617	133.75 ± 17.05	309	130.48 ± 15.48	308	136.76 ± 17.94	< 0.001
DBP, mmHg	617	87.47 ± 12.10	309	84.96 ± 11.59	308	89.70 ± 12.43	< 0.001
HR, bpm	617	81.19 ± 13.01	309	79.07 ± 12.06	308	83.33 ± 13.58	< 0.001
FPG, mmol/L	617	5.70 (5.24,6.79)	309	5.37 (5.07,5.88)	308	6.41 (5.60,8.36)	< 0.001
Fasting insulin, pmol/ml	580	214.95 (147.70, 324.70)	291	195.80 (132.90,282.35)	289	246.60 (156.80, 370.60)	< 0.001
HbA1c, %	607	5.80 (5.50,6.40)	303	5.60 (5.40,6.00)	304	6.10 (5.70,7.40)	< 0.001
TC, mmol/L	617	4.73 (4.21,5.40)	309	4.42 (4.04,4.96)	308	5.12 (4.48,5.78)	< 0.001
TG, mmol/L	617	1.68 (1.21,2.36)	309	1.22 (0.98,1.45)	308	2.37 (1.90,3.20)	< 0.001
HDL-C, mmol/L	614	1.10 (0.97,1.24)	309	1.14 (1.00,1.28)	305	1.05 (0.94,1.18)	< 0.001
LDL-C, mmol/L	614	3.12 (2.63,3.61)	309	2.92 (2.54,3.32)	305	3.38 (2.79,3.88)	< 0.001
BUN, mmol/L	615	4.60 (3.80,5.40)	309	4.60 (3.85,5.40)	306	4.70 (3.70,5.40)	0.721
SCR, µmol/L	615	58.00 (50.00,67.00)	309	57.00 (50.00,64.00)	306	59.00 (50.00,70.00)	0.006
TyG index	617	8.97 (8.60,9.39)	309	8.60 (8.33,8.79)	308	9.39 (9.16,9.83)	< 0.001
HOMA-IR	580	7.51 (4.86,12.32)	291	6.23 (3.98, 9.24)	289	9.93 (5.87, 16.29)	< 0.001
Complications, %							
Hypertension	617	235 (38.09)	309	86 (27.83)	308	149 (48.38)	< 0.001
Diabetes mellitus	617	172 (27.88)	309	29 (9.39)	308	143 (46.43)	< 0.001
Dyslipidaemia	617	359 (58.18)	309	96 (31.07)	308	263 (85.39)	< 0.001

*BMI* body mass index, *BSA* body surface area, *SBP* systolic blood pressure, *DBP* diastolic blood pressure, *HR* heart rate, *FPG* fasting plasma glucose, *HbA1c* glycated haemoglobin, *TC* total cholesterol, *TG* triglyceride, *HDL-C* high-density lipoprotein, *LDL-C* low-density lipoprotein, *BUN* blood urea nitrogen, SCR: serum creatinine

The obese patients in the high TyG index group were older and had higher blood pressure, resting HR, FPG, fasting insulin, HbA1c, TC, TG, LDL-C levels and SCR (all *P* < 0.01); and had lower HDL-C levels (*P* < 0.001). Moreover, the high TyG index group had greater incidences of hypertension, diabetes mellitus and dyslipidaemia. No significant differences were observed in height, weight, BMI, BSA waist circumference or BUN between the high TyG index group and low TyG index group (all *P* > 0.05).

### Echocardiographic parameters (Table 2)

**Table 2:** Echocardiographic parameters of obese patients between low TyG index and high TyG index

Echocardiographic parameters	*n*	Total	*n*	Low TyG index	*n*	High TyG index	*P* value
Septal thickness, mm	617	9.97 ± 1.14	309	9.82 ± 1.09	308	10.12 ± 1.17	0.001
Posterior wall thickness, mm	617	9.78 ± 1.09	309	9.68 ± 1.02	308	9.88 ± 1.15	0.025
LV diameter, mm	617	48.98 ± 4.25	309	48.83 ± 4.20	308	49.13 ± 4.26	0.377
Relative wall thickness	617	0.40 ± 0.04	309	0.40 ± 0.04	308	0.40 ± 0.04	0.097
LV mass, g	617	175.42 ± 48.92	309	171.41 ± 44.93	308	179.45 ± 52.39	0.041
LV mass index, g/m^2^	617	79.12 ± 17.48	309	78.04 ± 15.47	308	80.20 ± 19.26	0.126
LVH, %	617	49(8)	309	29(9)	308	20(6)	0.184
LVEDV, ml	617	89.65 ± 27.18	309	90.04 ± 29.74	308	89.26 ± 24.37	0.721
LVESV, ml	617	34.29 ± 12.68	309	34.50 ± 13.74	308	34.08 ± 11.53	0.683
LVEF, %	617	62.14 ± 4.02	309	62.08 ± 3.85	308	62.19 ± 4.18	0.740
MAPSE, mm	617	14.93 ± 1.75	309	14.88 ± 1.70	308	14.98 ± 1.81	0.504
LAV, ml	617	56.65 ± 15.21	309	57.11 ± 15.09	308	56.12 ± 15.30	0.422
LAVI, ml/m^2^	617	26.71 ± 6.63	309	27.16 ± 6.62	308	26.27 ± 6.62	0.097
E, m/s	616	0.81 ± 0.16	309	0.82 ± 0.15	307	0.80 ± 0.16	0.088
A, m/s	616	0.71 ± 0.17	309	0.69 ± 0.15	307	0.72 ± 0.18	0.010
E/A	616	1.21 ± 0.42	309	1.23 ± 0.32	307	1.18 ± 0.49	0.076
e′, m/s	615	0.11 ± 0.02	309	0.11 ± 0.02	306	0.11 ± 0.05	0.068
E/e′	615	7.52 ± 1.90	309	7.30 ± 1.67	306	7.74 ± 2.09	0.004
LV GLS, %	615	– 19.30 ± 2.85	309	– 19.61 ± 2.62	306	– 19.00 ± 2.02	0.010
LV GLS< – 16%, %	615	96(16)	309	45(15)	306	51(17)	0.472
LV GLS< – 20%, %	615	336(55)	309	161(52)	306	175(57)	0.205

*LV* left ventricular, LAV: left atrial volume, *LVH* left ventricular hypertrophy, *LVEDV* left ventricular end-diastolic volume, *LVESV* left ventricular end-systolic volume, *LVEF* left ventricular ejection fraction, *MAPSE* mitral annular plane systolic excursion, *E* peak velocity during early diastole of the anterior mitral leaflet, *A* peak velocity during late diastole of the anterior mitral leaflet, *e′* peak early diastolic annular velocities using TDI by averaging the values at the septum and lateral positions, *GLS* global longitudinal strain

The septal thickness, posterior wall thickness, LV mass, A and E/e′ in the high TyG index group were significantly greater than those in the low TyG index group (all *P* < 0.05). The LV GLS was significantly impaired in the high TyG index group compared with that in the low TyG index group (*P* = 0.01). No significant differences were observed in the LV diameter, relative wall thickness, LV mass index, LVEDV, LVESV, LVEF, MAPSE, LAV, LAVI, E, E/A, or e′ between the two groups (all *P* > 0.05). There were no significant differences in the percentages of patients with LVH, LV GLS< – 16%, or LV GLS< -20% between the two groups (all *P* > 0.05).

### Correlation tests of potential risk factors for LV GLS (Table 3)

**Table 3:** Correlation tests of potential risk factors for LV GLS

Parameters	LV GLS	
	*r*	*P* value
Age, years	– 0.069	0.086
Sex, male	– 0.373	< 0.001
HR, bpm	0.211	< 0.001
BMI, kg/m^2^	0.363	< 0.001
SBP, mmHg	0.254	< 0.001
FPG, mmol/L	0.148	< 0.001
HbA1c, %	0.201	< 0.001
TC, mmol/L	0.029	0.468
TG, mmol/L	0.164	< 0.001
HDL-C, mmol/L	– 0.191	< 0.001
LDL-C, mmol/L	0.039	0.338
BUN, mmol/L	0.081	0.045
SCR, µmol/L	0.207	< 0.001
TyG index	0.177	< 0.001
LV mass, g	0.465	< 0.001
LVH	0.138	0.001

HR, BMI, SBP, FPG, HbA1c, TG, BUN, SCR, the TyG index, LV mass and LVH were positively correlated with LV GLS (all *P* < 0.05), while male sex and HDL-C were negatively correlated with LV GLS (all *P* < 0.001).

### Multivariable logistic regression analysis of parameters associated with the TyG index and an LV GLS < 20% (Table 4; Fig. 2)

**Table 4:** Multivariable logistic regression analysis of parameters associated between TyG index and LV GLS < 20%

	OR	95%CI	*P* value
Model 1	1.692	1.329–2.155	< 0.001
Model 2	1.471	1.130–1.914	0.004
Model 3	1.386	1.052–1.825	0.020
Model 4	1.533	1.054–2.229	0.025
Model 5	1.520	1.040–2.221	0.031

Model 1 crude model

Model 2 adjusted for age and sex

Model 3 adjusted for model 2 covariates + BMI and HR

Model 4 adjusted for model 3 covariates + hypertension, diabetes mellitus, dyslipidaemia, blood urea nitrogen, and serum creatinine

Model 5 adjusted for model 4 covariates + LV mass and LVH

**Fig. 2:**
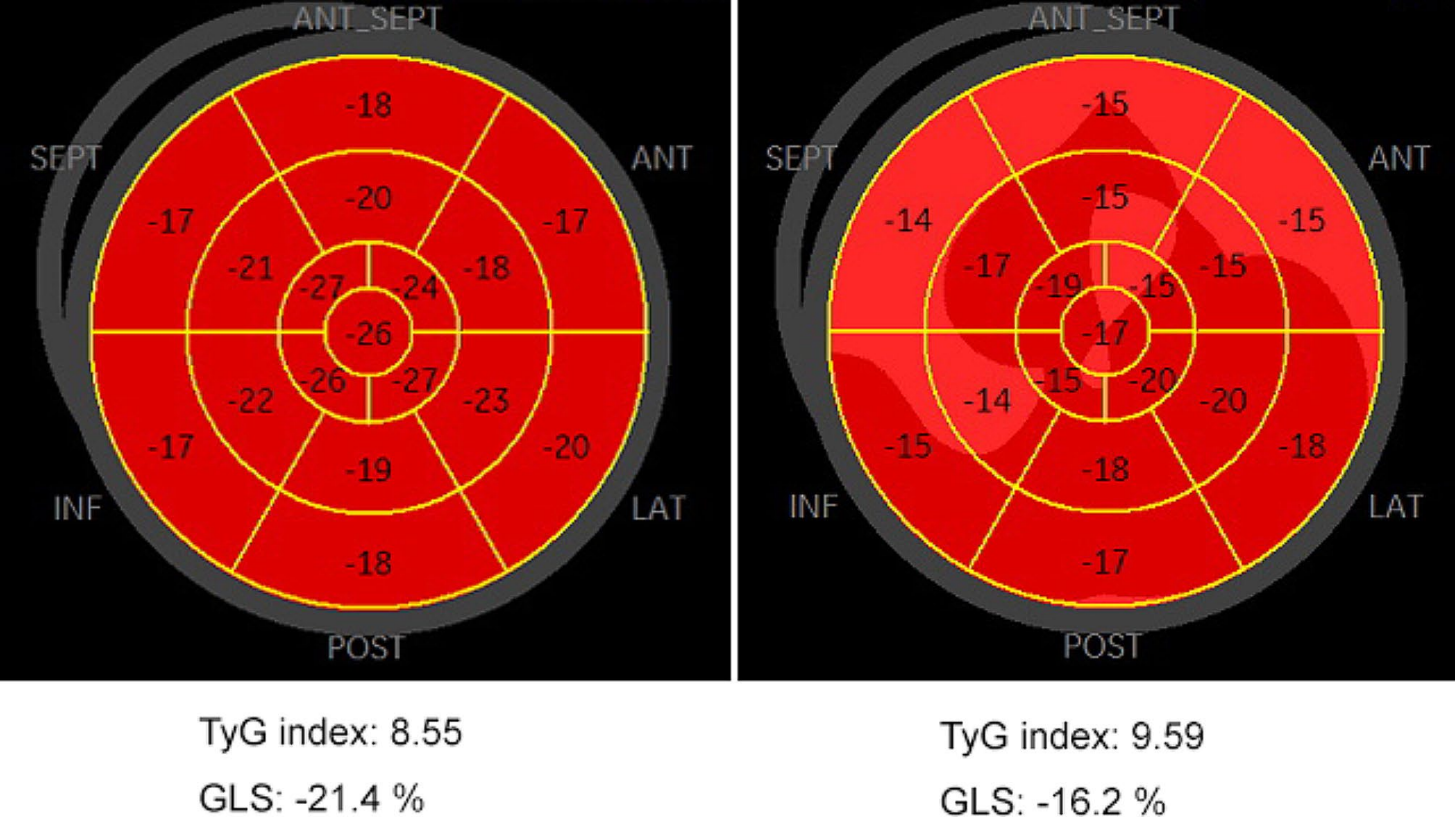
Bull′s eyes diagram showing the LV global longitudinal strain (GLS) and the related value of the TyG index

Variables for adjustment were selected according to two criteria: (1) variables with *P* < 0.05 in the correlation tests and (2) variables that were considered confounders based on the literature and clinical judgement.

Model 1 was an unadjusted model, and a correlation between the TyG index and LV GLS < 20% existed (OR: 1.692, 95% CI 1.329–2.115, *P* < 0.001). After age and sex adjustment, a correlation between the TyG index and LV GLS < 20% remained (OR: 1.471, 95% CI 1.130–1.914; *P* = 0.004). With further adjustment for confounders of age, sex, BMI and HR, a correlation between the TyG index and LV GLS < 20% still existed (OR: 1.386, 95% CI 1.052–1.825, *P* = 0.020). After adjusting for sex, age, BMI, HR, hypertension, diabetes mellitus, dyslipidaemia, blood urea nitrogen, and serum creatinine, the relationships between the TyG index and LV GLS < 20% still existed (OR: 1.533, 95% CI 1.054–2.229; *P* = 0.025). After adjusting for sex, age, BMI, HR, hypertension, diabetes mellitus, dyslipidaemia, blood urea nitrogen, serum creatinine, LV mass and LVH, the TyG index remained an independent risk indicator related to an LV GLS < 20% (OR: 1.520, 95% CI 1.040–2.221; *P* = 0.031).

### Intraobserver and interobserver variability

Intraobserver and interobserver variabilities were calculated by the intraclass correlation coefficient (ICC). LV GLS exhibited excellent intraobserver correlations with an ICC of 0.984 (95% CI 0.959–0.994) and interobserver correlations with an ICC of 0.980 (95% CI 0.950–0.992).

## Discussion

We found that as the TyG index increased in obese patients, the absolute value of the LV GLS decreased. Multivariable logistic regression analysis revealed that the TyG index was independently associated with the LV GLS, indicating that an increase in the TyG index seems to provide more reference value for early clinical detection of subclinical LV myocardial systolic dysfunction in obese patients.

Recent studies have widely used the TyG index as a marker of insulin resistance. A higher TyG index may be independently associated with subclinical LV dysfunction in patients with coronary heart disease [6] or acute coronary syndrome [20] and is likely to be associated with a greater risk of arterial stiffness [21] or carotid atherosclerosis progression [22]. Selvi NMK et al. reported that the TyG index can be used as a simple and inexpensive alternative for assessing glycaemic control in patients with diabetes [23]. In patients with heart failure with preserved ejection fraction (HFpEF), a high TyG index is also associated with an increased risk of mortality and rehospitalization [24]. The TyG index is also associated with chronic kidney disease [25], stroke [26], diabetes [27], prediabetes [28], hypertension [29], etc.

At present, whether the TyG index is related to subclinical LV systolic dysfunction in obese patients has not been investigated. Therefore, this study aimed to provide more reference values in this field and innovatively introduced the LV GLS parameter, which is helpful for conveniently and quickly evaluating LV systolic dysfunction in obese patients in the early stage, preventing the worsening of myocardial fibrosis and improving the prognosis.

In this study, we found that an increase in the TyG index may be associated with subclinical LV systolic dysfunction in obese patients. As calculated from TG and FPG, the TyG index is closely related to insulin resistance. Insulin resistance refers to a reduced ability of insulin to stimulate glucose utilization, while glucose tolerance remains normal [30]. Obesity is clearly the most common cause of insulin resistance [31]. Obesity is characterized by chronic tissue activation of inflammation [32], and an important relationship between systemic inflammation caused by maladaptive adipose tissues and insulin resistance in individuals with obesity has been suggested [33]. Cardiac fibrosis is strongly associated with obesity and metabolic dysfunction [34]. The TyG index and cardiac fibrosis have been demonstrated to be positively correlated. Considering the above mechanisms, we found that the TyG index may be associated with subclinical LV systolic dysfunction in obese patients.

Correlation analysis of the potential risk factors revealed that female sex, HR, BMI, SBP, FPG, HbA1c, TG, HDL-C, BUN, SCR, the TyG index, LV mass and LVH were factors related to LV GLS in obese patients. Obesity may influence LV geometry substantially more in women than in men because of hypertension or sex differences in biological factors specifically associated with visceral fat [35, 36]. At present, there are no studies on the mechanism of this phenomenon, which may be related to differences in metabolism and hormone levels between the sexes. In obese patients with hypertension, an increase in long-term afterload will cause LV myocardial hypertrophy and fibroblast proliferation, promote the occurrence and development of myocardial fibrosis [37], and cause greater impairment of LV systolic function. In obese patients with diabetes mellitus, hyperinsulinaemia or insulin resistance will affect homeostasis, and an elevated blood glucose will have toxic effects on myocardial cells [38], which will aggravate LV systolic dysfunction in obese patients. The toxic effect of dyslipidaemia on cardiomyocytes has been described in existing studies [39]; moreover, obese patients with hypertriglyceridaemia will have greater impairment of LV systolic function than obese patients with normal blood lipids. The multivariable logistic regression analysis was adjusted according to these potential risk factors. An increase in the TyG index was independently correlated with impaired LV GLS (*P* = 0.031). This seems to support the value of the early identification of subclinical LV myocardial systolic dysfunction in obese patients in clinical practice. After identifying subclinical LV myocardial systolic dysfunction in obese patients, the BMI can be reduced through diet and exercise and, if necessary, medication or sleeve gastrectomy [40]. For obese patients with hypertension, diabetes, renal dysfunction or dyslipidaemia, sodium-glucose cotransporter protein-2 (SGLT-2) inhibitors and other drugs [41] should be used. All of these factors can help obese patients prevent further impairment of their LV myocardial systolic function, stopping it from eventually developing intoirreversible myocardial fibrosis.

## Conclusions

From this research, we concluded that an increase in the TyG index is independently associated with subclinical LV systolic dysfunction in obese patients.

### Limitations

First, this study was conducted at a single centre, which may limit the generalizability of the findings to broader populations. Multicentre studies involving diverse demographic and geographic populations could enhance the external validity of the results. Second, long-term follow-up data on clinical outcomes such as cardiovascular events or mortality were lacking. Third, all of these obese patients underwent sleeve gastrectomy, but whether their LV systolic function improved requires follow-up.

## Data Availability

No datasets were generated or analysed during the current study. The datasets used and analyzed during the current study are available from the corresponding author on reasonable request.

## Abbreviations

TyG index: triglyceride-glucose index
T2DM: type 2 diabetes mellitus
BMI: body mass index
BSA: body surface area
SBP: systolic blood pressure
DBP: diastolic blood pressure
HR: heart rate
FPG: fasting plasma glucose
HbA1c: glycated haemoglobin
TC: total cholesterol
TG: triglyceride
HDL-C: high-density lipoprotein
LDL-C: low-density lipoprotein
BUN: blood urea nitrogen
SCR: serum creatinine
LVd: left ventricular diameter in the end-diastolic period
LVEDV: left ventricular end-diastolic volume
LVESV: left ventricular end-systolic volume
LVEF: left ventricular ejection fraction
MAPSE: mitral annular plane systolic excursion
GLS: global longitudinal strain
